# Supplementary material for: Monitoring changes in vitamin D levels during the COVID-19 pandemic with routinely-collected laboratory data
Source: Nat Commun. 2025 Oct 2;16:8772. doi: 10.1038/s41467-025-64192-6 (PMC12491433; doi:10.1038/s41467-025-64192-6)
Supplement: Supplementary file 1 — Supplementary Information [file 41467_2025_64192_MOESM1_ESM.pdf]

Supplements

Supplementary Figures

S1 Mean vitamin D levels over time 44

S2 Early vs. late pandemic 45

S3 Year-to-year changes in vitamin D levels 46

S4 Forest plot of pandemic effect by region. 62

Supplementary Tables

S1 Vitamin D levels (median and interquartile range) 47

S2 Characteristics of propensity score-matched sample 48

S3 Machine learning subgroup analysis stratified by age, gender, and season 49

S4 Sensitivity analysis results for key covariates 52

S5 Balance diagnostics for covariates before and during the pandemic 53

S6 Categorical breakdown of balance diagnostics 54

S7 Regression analysis for the mean differences in vitamin D levels 56

S8 Regression analysis for vitamin D deficiency 57

S9 Mean vitamin D levels across different types of regions 59

S10 Deficiency rates of vitamin D by different types of regions 60

S11 Linear and logistic regression with time trend 63

Supplementary Materials

A Sensitivity analysis **50**

B Regression analysis **55**

C Geographic analysis **58**

C.1 Descriptive analyses 58

C.2 Propensity-score matching 61

C.3 Causal forest 62

D Analysis with time trend **63**

E Longitudinal analysis **64**

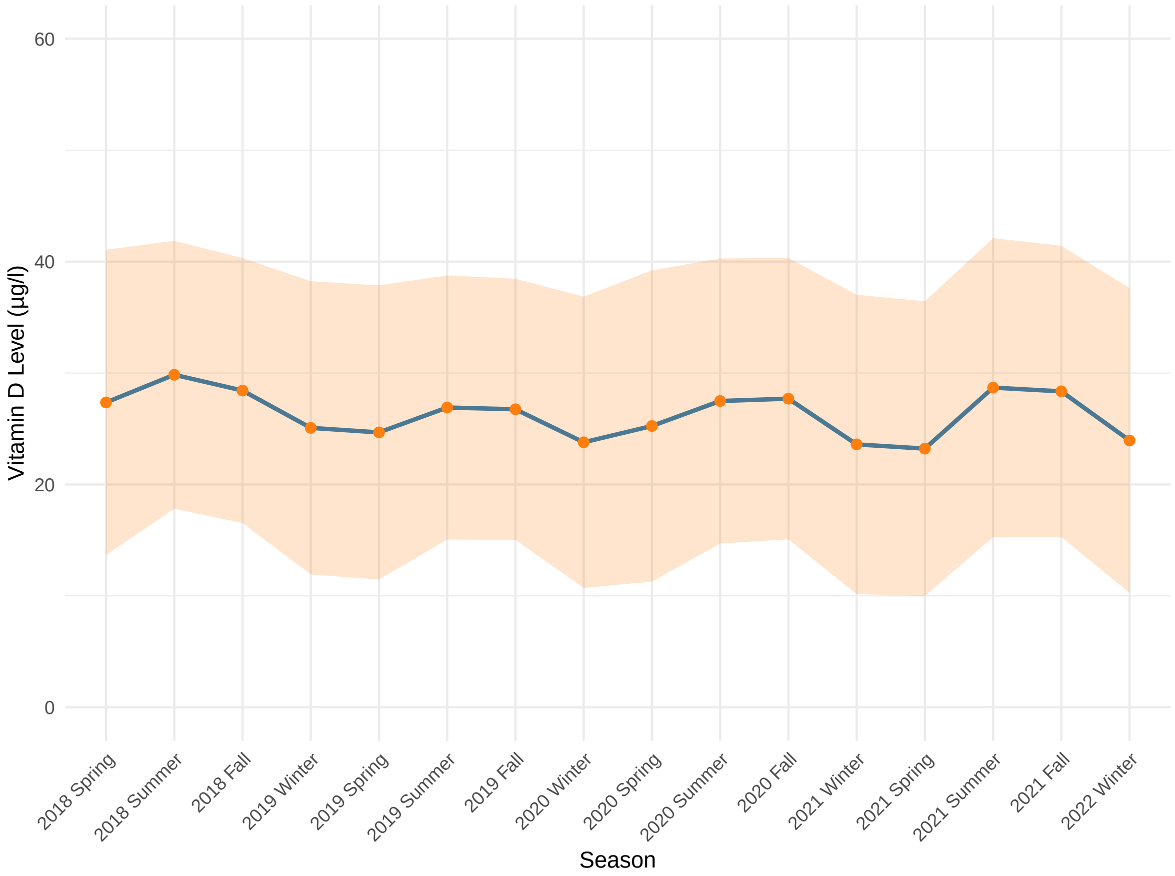

Figure S1: **Mean vitamin D levels over time.** Mean vitamin D levels (µg/l) across the study period from Winter 2018 to Winter 2022. Each point represents the mean vitamin D level for the entire study population for a specific season, while a shaded area denotes the one standard deviation around the mean. Source data are provided as a Source Data file.

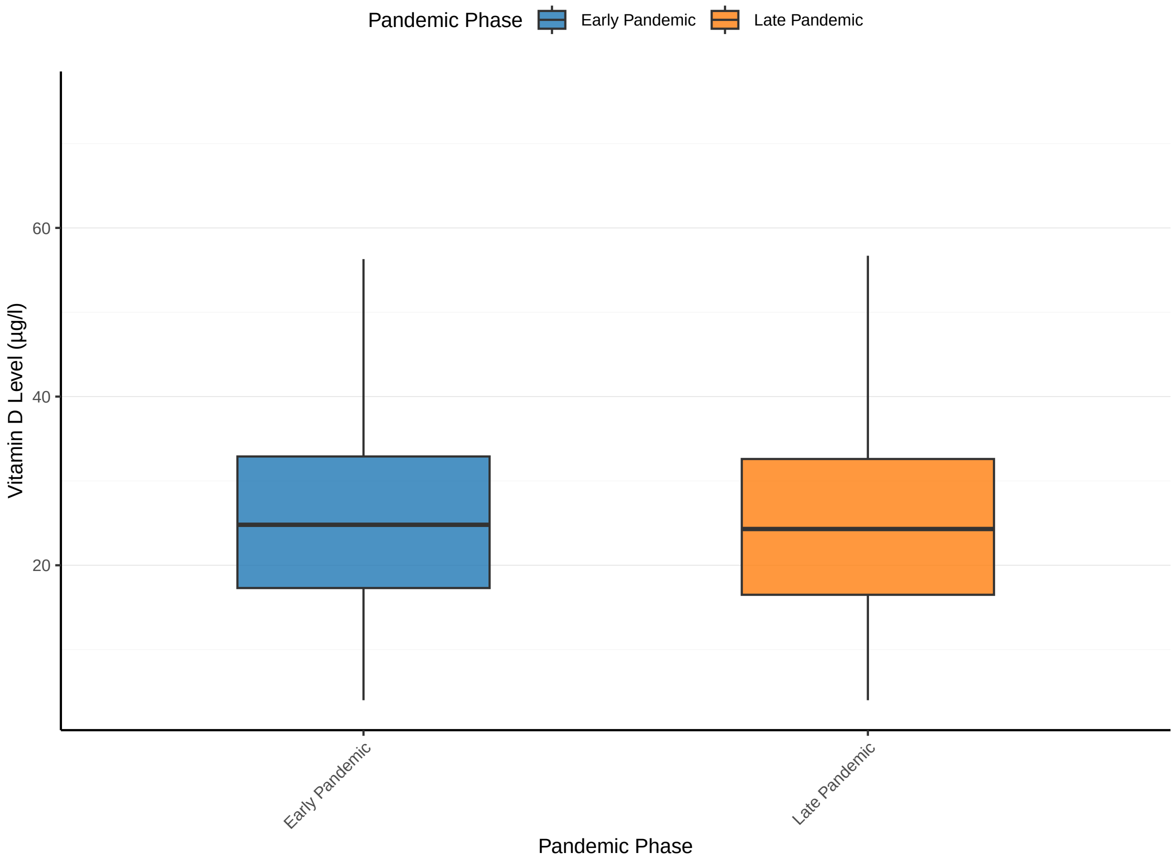

Figure S2: **Mean vitamin D levels during early vs. late pandemic.** Mean vitamin D levels (µg/l) in the early pandemic phase (March 2020 – August 2020) compared to the late pandemic phase (September 2021 – February 2022) using a t-test (***: p-value < 0.001).

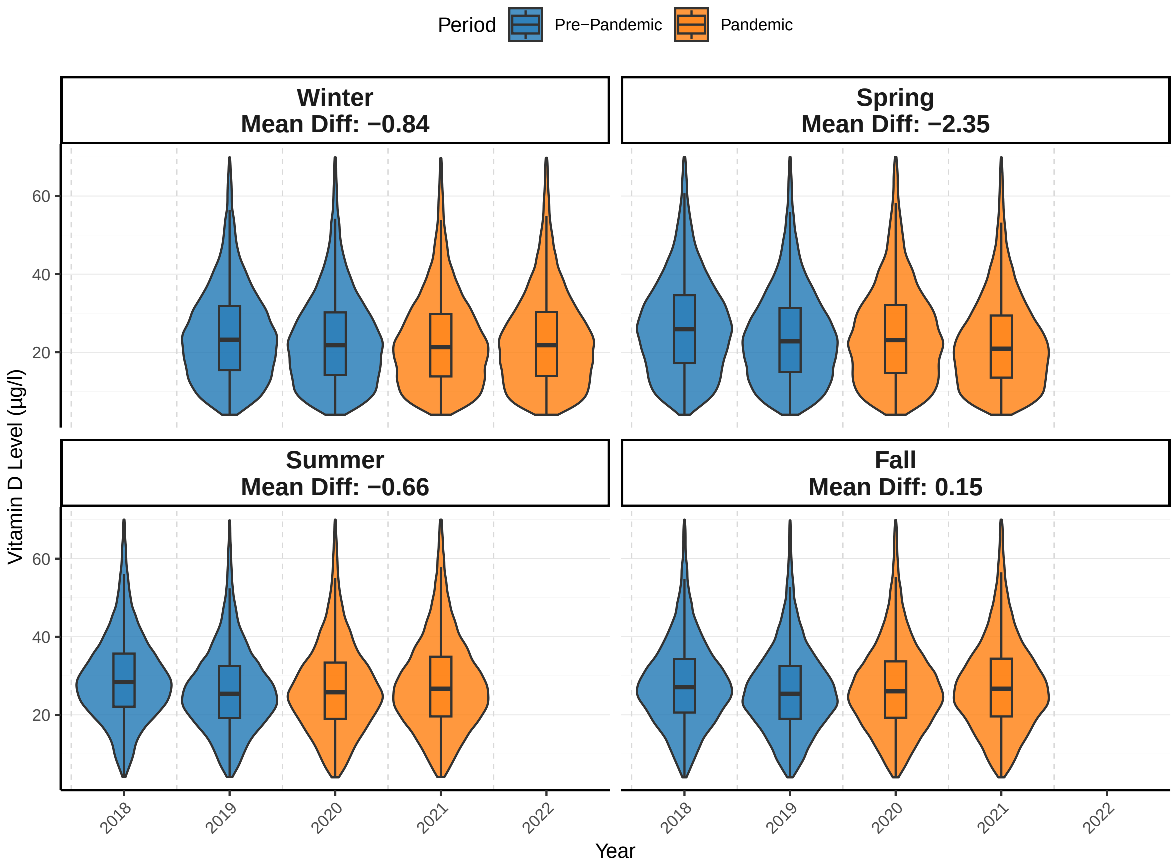

Figure S3: **Year-to-year changes in vitamin D levels.** Violin plots display the distribution of vitamin D levels for each season and year before (March 2018 – February 2020) and during (March 2020 – February 2022) the pandemic. The black box inside each violin represents the interquartile range (IQR), with the horizontal line marking the median. The thin whiskers extend to 1.5 times the IQR. The black point and error bars indicate the mean and one standard deviation (SD) around the mean. The mean differences between pre-pandemic and pandemic periods are reported separately for each season.

Table S1: **Vitamin D levels (median and interquartile range)** Comparison of median vitamin D levels in serum (in µg/l) between pre-pandemic and pandemic periods across different subgroups stratified by age, gender, and season. Statistical comparisons are based on Wilcoxon rank-sum tests. IQR: interquartile range value.

	**Pre-pandemic**	**Pandemic**	**p-value**
**Total**			
**By age, years (median, [IQR])**			
18–39	24.2 [17.0–31.6]	23.3 [16.1–31.1]	$p = 5.73 \times 10^{-19}$
40–59	25.2 [18.2–33.0]	24.2 [17.0–32.4]	$p = 2.22 \times 10^{-37}$
60+	26.3 [18.1–34.7]	25.2 [16.6–34.3]	$p = 3.35 \times 10^{-35}$
**By gender (median, [IQR])**			
Females	26.2 [18.7–34.3]	25.0 [17.4–33.5]	$p = 1.18 \times 10^{-74}$
Males	23.9 [16.5–31.8]	23.0 [15.3–31.3]	$p = 8.97 \times 10^{-31}$
**By season (median, [IQR])**			
Winter (Dec – Feb)	22.7 [14.9–31.3]	21.7 [13.9–30.4]	$p = 7.41 \times 10^{-25}$
Spring (Mar – May)	24.6 [16.2–33.6]	21.7 [14.0–30.7]	$p = 3.24 \times 10^{-163}$
Summer (Jun – Aug)	27.3 [20.9–34.6]	26.4 [19.4–34.5]	$p = 1.14 \times 10^{-19}$
Fall (Sep – Nov)	26.5 [19.9–33.7]	26.5 [19.5–34.4]	$p = 1.14 \times 10^{-19}$

Table S2: **Characteristics of propensity score-matched sample.** Characteristics for gender and age groups before and during the pandemic as well as mean vitamin D levels and vitamin D deficiency rate across all groups. Sample size before matching: 292,187 unique individuals. Sample size after matching: 267,380 unique individuals. Number of unique individuals lost due to the matching process: 24,807 (8.5%). Statistical testing was done based on two-sided Pearson's χ^2-tests for categorical data and two-sided Welch's t-tests for all numerical data, respectively. Exact p-values are provided.

	**Pre-pandemic**	**Pandemic**	p**-value**
	(*N*=133,410)	(*N*=133,410)	
**Age, years (*N*, [%])**			
18–39	34,204 [25.6]	34,204 [25.6]	1.000
40–59	49,535 [37.1]	49,535 [37.1]	1.000
60+	49,671 [37.2]	49,671 [37.2]	1.000
**Gender (*N*, [%])**			
Females	84,880 [63.6]	84,880 [63.6]	1.000
Males	48,530 [36.4]	48,530 [36.4]	1.000
**Season (*N*, [%])**			
Winter (Dec – Feb)	33,878 [25.4]	33,878 [25.4]	1.000
Spring (Mar – May)	32,028 [24.0]	32,028 [24.0]	1.000
Summer (Jun – Aug)	31,222 [23.4]	31,222 [23.4]	1.000
Fall (Sep – Nov)	36,282 [27.2]	36,282 [27.2]	1.000
**Outcomes**			
Vitamin D levels, µg/l (mean, [SD])	26.48 [12.7]	25.99 [13.4]	$p = 1.11 \times 10^{-22}$
Vitamin D deficiency rate (n, [%])	42,188 [31.6]	46,926 [35.2]	$p = 3.02 \times 10^{-84}$

Table S3: **Machine learning subgroup analysis stratified by age, gender, and season.** Estimated change in vitamin D levels in μg/l for each covariate sub-category and the corresponding confidence intervals. For example, for the age group 40–59, a negative treatment effect of close to -1 indicates that the pandemic group had an average reduction of close to 1 μg/l in vitamin D serum levels compared to the pre-pandemic group. This suggests that, for individuals aged 40–59, the intervention (in this study: the onset of the pandemic) is associated with a confounder-adjusted decrease in vitamin D levels.

**Category**	**Estimated change**	**Lower 99% CI**	**Upper 99% CI**	**Lower 95% CI**	**Upper 95% CI**
Age Group: 18–39	−0.6358	−0.8693	−0.4023	−0.8135	−0.4581
Age Group: 40–59	−0.7250	−0.9281	−0.5219	−0.8795	−0.5704
Age Group: 60+	−0.6575	−0.8658	−0.4491	−0.8160	−0.4989
Females	−0.7474	−0.9045	−0.5903	−0.8669	−0.6279
Males	−0.5572	−0.7573	−0.3571	−0.7095	−0.4050
Season: Spring (Mar – May)	−2.0641	−2.3179	−1.8103	−2.2572	−1.8710
Season: Summer (Jun – Aug)	−0.4208	−0.6739	−0.1677	−0.6134	−0.2283
Season: Fall (Sep – Nov)	0.3998	0.1698	0.6298	0.2248	0.5748
Season: Winter (Dec – Feb)	−0.6034	−0.8563	−0.3506	−0.7958	−0.4111

Supplement A Sensitivity analysis

We conducted a causal sensitivity analysis using the `sensemakr` package [58] to address the possibility of unobserved confounding. Thereby, we can ensure that, even in the presence of unobserved confounding, our coefficient estimates cannot be explained away and thus remain robust. Specifically, we compute the robustness value (RV), which quantifies the minimum strength of association that unobserved confounding would need to have, both with the treatment and with the outcome, to alter our conclusions [58]. The RV indicates how strong a potential unobserved confounder would need to be to (a) bring our coefficient estimates to zero ($RV_{q=1}$) and (b) render the coefficient estimates no longer significant at the $\alpha = 0.01$ level ($RV_{q=1,\alpha=0.01}$). Since the `sensemakr` package works directly with linear regression models, we approximated the causal forest results with a linear regression framework where the vitamin D serum level is the dependent variable (DV). The results are in Table S4.

For the intervention variable (COVID-19 pandemic), the robustness values indicate that (a) an unobserved confounder would need to explain at least 2.42% of the residual variance in the association with intervention or outcome to fully explain away the observed effect ($RV_{q=1} = 2.42\%$) and (b) would need to explain 2.07% of the residual variance to make the effect statistically non-significant at the $\alpha = 0.01$ level ($RV_{q=1,\alpha=0.01} = 2.07\%$).

The robustness values exceed the explanatory power of an unobserved confounder as strong as the observed covariates (age, gender, and season), as shown in the sensitivity analysis results (Table S4). For example, we can compare the robustness value against the maximum explained residual variance due to an unobserved confounder as strong as the covariate "age group". For the latter, the partial R^2 of a confounder with the intervention is 0.28% and the partial R^2 of a confounder with the intervention is 0.34%. Both values are smaller than the thresholds from the robustness value. Therefore, an unobserved confounder would need to be considerably stronger than the covariate "age group" (or any other covariate) to nullify our findings. Based on these

results, our conclusions regarding the effect of the COVID-19 pandemic on vitamin D levels are robust to reasonable amounts of potentially unobserved confounding.

Table S4: **Sensitivity analysis results for key covariates.** The covariates age group, gender, and season were used as benchmarks to compare the strength of association that an unobserved confounder would need to have to explain away the association between both the pandemic intervention and the vitamin D reduction. The benchmarks were selected because they are known and important drivers of heterogeneity in vitamin D levels across the broader population. To make the treatment effect statistically non-significant at the $\alpha = 0.01$ level, an unobserved confounder would need to explain 2.07% of the residual variance in the association with intervention or outcome ($RV_{q=1,\alpha=0.01}$). For the treatment effect to be reduced to zero, an unobserved confounder would need to explain at least 2.42% of the residual variance in the association with intervention or outcome ($RV_{q=1}$). Hence, ideally, explained variance due to an unobserved confounder as strong as the benchmarks should be lower than the robustness values. As desired, this is observed. We thus report the corresponding explained variance of a confounder as strong as the benchmark based on the partial R^2 with intervention and partial R^2 with outcome. Both values are smaller than the robustness values, implying that a confounder as strong as age, gender, or season would not make the results non-significant (due to $< RV_{q=1,\alpha=0.01}$) or go to zero (due to $< RV_{q=1}$). Hence, a potential unobserved confounder must be substantially more important than any of the benchmarks. We also report the adjusted coefficient (adj. coef.) and the adjusted standard error (adj. s.e.) for a hypothetical setting reflecting the pandemic intervention and an unobserved confounder equally as strong as the association between the observed benchmark (i.e., age, gender, season) and the vitamin D levels. The adjusted estimates for each benchmark confirm that a reasonable confounder (i.e., a confounder as strong as age, gender, or season) would not invalidate the observed negative treatment effect of the pandemic on vitamin D levels.

**Benchmark**	Partial R^2 with intervention $R^2_{D\sim Z\|\mathbf{X}}$	Partial R^2 with outcome $R^2_{Y\sim Z\|\mathbf{X},D}$	**Adj. coef**	**Adj. s.e.**
Age	0.28%	0.34%	−0.593	0.0480
Gender	0.01%	0.65%	−0.614	0.0479
Season	0.01%	1.48%	−0.603	0.0477

Table S5: **Balance diagnostics for covariates before and during the pandemic.** We calculated the weighted absolute standardized mean difference (ASMD) for the three key covariates (age group, gender, and test month) between the pre-pandemic and pandemic study populations. ASMD values close to 0 indicate well-balanced groups, implying that propensity scores are well-calibrated. All ASMD values remained below the recommended threshold of 0.1 [59], confirming strong covariate balance after weighting. The mean values for each covariate in both study populations are also reported. The pre-pandemic mean and pandemic mean columns represent the average encoded values of age group, gender, and test month in the respective study populations. Age groups were encoded as 1 = 18—39 years, 2 = 40-–59 years and 3 = 60+ years. Gender was encoded as 1 = female and 2 = male. Test month was encoded chronologically (e.g., 1 = January, 2 = February, etc.).

**Covariate**	**Pre-pandemic mean**	**Pandemic mean**	**Weighted ASMD**	**Threshold**
Age Group	2.170026	2.080980	0.0000	Balanced, <0.1
Gender	1.357013	1.365407	0.517×10^{-19}	Balanced, <0.1
Test Month	6.242906	6.524429	-0.405×10^{-16}	Balanced, <0.1

Table S6: **Categorical breakdown of balance diagnostics.** Weighted absolute standardized mean differences (ASMD) are reported for each level of the covariates age group, gender, and test month. A value below 0.1 indicates adequate balance. All ASMDs were well below this threshold. The variance ratio (treated / control) of the propensity scores was 0.935, indicating acceptable balance between groups.

Covariate Level	Weighted ASMD
Age 18–39	0.00005
Age 40–59	0.00015
Age 60+	−0.00020
Male	0.00005
January	0.00042
February	0.00008
March	−0.00030
April	−0.00111
May	−0.00009
June	0.00020

Supplement B Regression analysis

We conducted two additional regression-based analyses. Specifically, we performed both a multivariate logistic regression to link the pandemic to (i) the changes in continuous serum vitamin D levels (via a linear regression) and (ii) the odds of vitamin D deficiency (via a logistic regression). The linear regression model (Table S7) estimates the adjusted mean differences in serum vitamin D concentrations across the same covariates. The results of the logistic regression model (Table S8) quantify how the odds of vitamin D deficiency changed during the pandemic and across demographic and seasonal strata. Both models demonstrate statistically significant associations between the pandemic period and vitamin D outcomes, and also confirm established patterns such as seasonal variation and gender differences. These regression results corroborate the findings from our main paper, while providing additional interpretability.

All models were implemented in R using the `glm` and `lm` functions for logistic and linear regression, respectively [60].

Table S7: **Regression analysis for the mean differences in vitamin D levels.** Results from a multivariate linear regression model with serum vitamin D concentration as the outcome. Estimated via ordinary least squares (OLS). The model adjusts for period, age group, gender, and season. Estimates represent the mean difference in vitamin D levels (ng/ml) compared to the reference categories. Individuals were $\geq$18 years; no <18 category. All coefficients are statistically significant ($p < 0.001$).

**Variable**	**Estimate (ng/ml)**	**99% CI (Lower)**	**99% CI (Upper)**
Intercept	23.89	23.89	24.25
Pandemic period (vs Pre-pandemic)	−0.67	−0.80	−0.55
Pre-pandemic (ref)	—	—	—
Age 40–59 (vs 18–39)	1.32	1.16	1.48
Age 60+ (vs 18–39)	1.98	1.82	2.14
Age 18–39 (ref)	—	—	—
Male (vs Female)	−2.17	−2.30	−2.04
Female (ref)	—	—	—
Spring (Mar – May) (vs Winter (Dec – Feb))	0.93	0.76	1.10
Summer (Jun – Aug) (vs Winter (Dec – Feb))	4.16	3.98	4.34
Fall (Sep – Nov) (vs Winter (Dec – Feb))	3.63	3.46	3.80
Winter (Dec – Feb) (ref)	—	—	—

Table S8: **Regression analysis for vitamin D deficiency.** Results from a multivariate logistic regression model evaluating the association between pandemic period and vitamin D deficiency as odds ratio. Odds ratios (ORs) > 1 indicate increased odds of deficiency, and ORs < 1 indicate reduced odds, relative to the reference categories. Individuals were $\geq$18 years; no <18 category. All estimates are statistically significant ($p < 0.001$).

**Variable**	**Odds Ratio**	**99% CI (Lower)**	**99% CI (Upper)**
Intercept	0.681	0.662	0.701
Pandemic period (vs Pre-pandemic)	1.211	1.186	1.237
Pre-pandemic (ref)	—	—	—
Age 40–59 (vs 18–39)	0.833	0.811	0.856
Age 60+ (vs 18–39)	0.809	0.787	0.830
Age 18–39 (ref)	—	—	—
Male (vs Female)	1.416	1.387	1.447
Female (ref)	—	—	—
Spring (Mar – May) (vs Winter (Dec – Feb))	0.891	0.867	0.916
Summer (Jun – Aug) (vs Winter (Dec – Feb))	0.433	0.420	0.446
Fall (Sep – Nov) (vs Winter (Dec – Feb))	0.468	0.455	0.482
Winter (Dec – Feb) (ref)	—	—	—

Supplement C Geographic analysis

C.1 Descriptive analyses

To explore potential geographic variation in vitamin D status, we stratified the study population by different types of regions, namely, categorized into cities, rural areas, and suburbs based on the Degree of Urbanisation classification by Eurostat [61]. Due to data protection, postal code information was not made available to us. The data provider could, however, include the information on the Degree of Urbanization by linking the postal code to the DEGURBA urbanity type. If a clear attribution of a postal code to exactly one urbanity type was not possible due to overlapping boundaries, the postal code was attributed to the urbanity type in which it had the largest geographical area (Geographic attribution and area calculation done with QGIS version 3.34). Please note that more granular regional analyses (e.g., at the district or address level) were not feasible due to constraints imposed by the ethical oversight board, as they would increase the risk of re-identification.

Overall, the majority of individuals resided in urban areas, with over 173,000 individuals assigned to cities, followed by nearly 90,000 in suburbs, and approximately 41,000 in rural areas. Across all regions, mean vitamin D levels declined during the pandemic. In cities, levels decreased from 26.74 µg/L before the pandemic to 25.98 µg/L during the pandemic. In suburbs, the average dropped from 27.41 µg/L to 26.25 µg/L, and in rural areas, from 27.57 µg/L to 26.48 µg/L. These within-region differences comparing before pandemic and pandemic periods were statistically significant in all cases ($p < 0.001$), as shown in Table S9.

Vitamin D deficiency rates followed a similar pattern. Prior to the pandemic, deficiency was most common in cities (30.8%), followed suburbs (28.9%) and rural areas (28.6%). During the pandemic, deficiency rates increased across all regions, with the sharpest rise observed in suburbs (to 34.5%) and cities (to 34.9%) (Table S10). All observed changes were statistically significant ($p < 0.001$).

Table S9: **Mean vitamin D levels across different types of regions.** Comparison of mean serum vitamin D levels (in µg/l) before and during the pandemic across different types of regions (city, rural, and suburb) based on the Degree of Urbanisation classification by Eurostat [61]. Statistical comparisons are based on two-sided Welch's t-tests. Exact p-values are provided.

**Region**	**Pre-pandemic**	**Pandemic**	**p-value**
City	26.40	25.82	$p = 7.72 \times 10^{-20}$
Rural area	27.12	26.30	$p = 1.52 \times 10^{-9}$
Suburb	26.99	26.09	$p = 1.26 \times 10^{-23}$

Table S10: **Deficiency rates of vitamin D by different types of regions.** Comparison of deficiency rates before and during the pandemic across different types of regions (city, rural, and suburb) based on the Degree of Urbanisation classification by Eurostat [61]. Deficiency is defined as vitamin D serum concentration below 20 µg/l. Statistical comparisons are based on two-sided Pearson's χ^2-tests. Exact p-values are provided.

**Region**	**Pre-pandemic**	**Pandemic**	**p-value**
City	28,144 [32.0%]	27,733 [35.5%]	$p = 5.37 \times 10^{-50}$
Rural area	6,231 [30.2%]	6,477 [34.3%]	$p = 2.51 \times 10^{-18}$
Suburb	13,594 [30.2%]	14,408 [35.1%]	$p = 9.05 \times 10^{-53}$

C.2 Propensity-score matching

We performed propensity score matching where we additionally included the two-digit postal code as regional covariate. For this, a matched study population of 130,641 individuals per period (pre-pandemic and pandemic) was obtained from an initial sample of 292,187 individuals (i.e., total matched sample size of 261,282 observations). This procedure excluded 30,905 individuals (10.6%) due to a lack of suitable matches. In the matched sample, the mean serum vitamin D level decreased from 26.47 µg/L (SD 12.71) before the pandemic to 25.99 µg/L (SD 13.44) during the pandemic. The vitamin D deficiency rate increased from 31.7% (41,432 individuals) to 35.2% (45,948 individuals). The standardized mean difference (SMD) for continuous serum vitamin D levels was -0.0369 (99% CI: -0.0467 to -0.0274), indicating a small but consistent reduction. For the binary deficiency outcome, the SMD was 0.0733 (99% CI: 0.0632 to 0.0834), suggesting a meaningful increase in deficiency prevalence following the onset of the pandemic.

C.3 Causal forest

Further, we again employed a causal forest analysis. Across all individuals, the average treatment effect (ATE) of the pandemic was estimated at -0.678 µg/L (99% CI: -0.802 to -0.554), corresponding to a mean decrease of 2.5% compared to the pre-pandemic average level of 26.68 µg/L. To assess whether the pandemic effect differed across geographic subgroups, we stratified the causal forest analysis by the regional category variable. The results are visualized in Supplementary Figure S4, which indicates a consistent reduction in vitamin D levels across all region types, with slightly more pronounced effects observed in suburb areas.

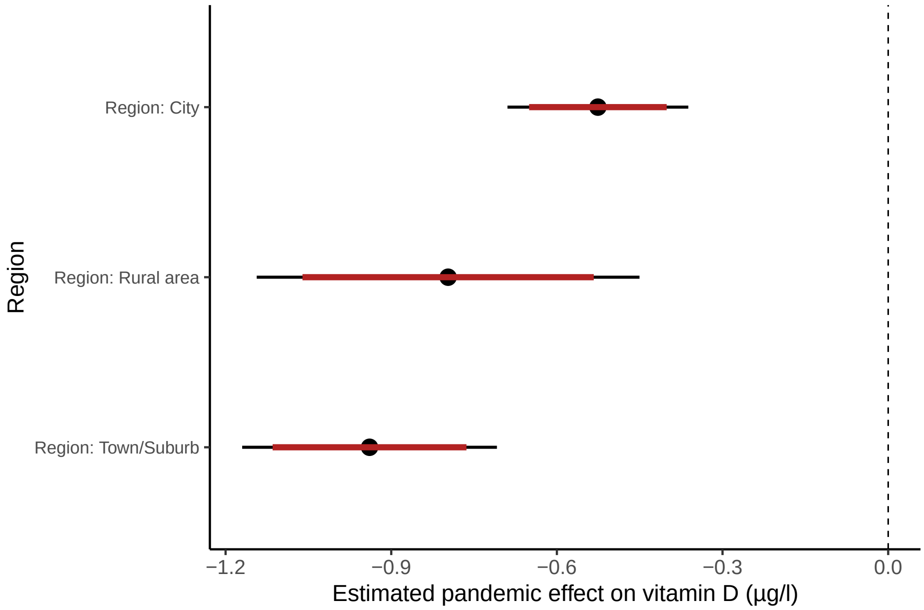

Figure S4: **Estimated pandemic effect on vitamin D levels by region.** Forest plot showing the treatment effect of the pandemic on serum vitamin D levels (µg/L), stratified by region (city, rural area, suburb) using a causal forest model. The plot displays point estimates with 95% and 99% confidence intervals. Source data are provided as a Source Data file.

Supplement D Analysis with time trend

To assess the presence of a linear time trend in test values, we performed a multivariate linear regression with the test result as the outcome and the number of months since March 2020 as a continuous predictor (negative values indicate pre-pandemic periods). The model includes additional controls for age group, gender, and season (see Supplementary Table S11, left). The analysis reveals a statistically significant negative association between time and test values ($\beta = -0.038$, 99% CI: $[-0.043; -0.034]$, $p < 0.001$), indicating a gradual decline of approximately 0.038 units per month over the study period and thus points to lower vitamin D levels during the pandemic.

To complement this analysis, we additionally estimated a logistic regression model using deficiency status (serum value < 20 µg/L) as a binary outcome (see Supplementary Table S11, right). This model revealed a statistically significant positive association between time and deficiency odds ($\beta = 0.009$, 99% CI: $[0.008; 0.010]$, $p < 0.001$), suggesting that the likelihood of deficiency slightly increased over time. All covariates, including age group, gender, and season, were significantly associated with both continuous test values and deficiency risk.

Table S11: **Regression analysis with time trend.** Estimated coefficients from linear and logistic regression analysis modeling the effect of time and covariates on test value and deficiency. The linear model uses the continuous test value as the dependent variable; the logistic model uses deficiency as the binary dependent variable. Coefficients are shown with 99% confidence intervals (CIs). The p-values are derived from t-tests (linear model) and Wald z-tests (logistic model) for each coefficient.

**Covariate**	**Linear model (Estimate [99% CI])**	**Logistic model (Estimate [99% CI])**	**p-values (Linear; Logistic)**
(Intercept)	23.91 [23.75; 24.06]	-0.33 [-0.35; -0.30]	$p < 1 \times 10^{-308}$; $p = 3.82 \times 10^{-161}$
Time (months)	-0.038 [-0.043; -0.034]	0.009 [0.008; 0.010]	$p < 3.02 \times 10^{-111}$; $p = 9.94 \times 10^{-207}$
Age 40–59	1.29 [1.11; 1.47]	-0.18 [-0.20; -0.16]	$p = 1.03 \times 10^{-105}$; $p = 1.18 \times 10^{-77}$
Age 60+	1.93 [1.76; 2.06]	-0.21 [-0.23; -0.19]	$p = 1.13 \times 10^{-235}$; $p = 2.85 \times 10^{-107}$
Male	-2.17 [-2.31; -2.02]	0.35 [0.33; 0.37]	$p < 1 \times 10^{-308}$; $p < 1 \times 10^{-308}$
Spring (Mar – May)	0.58 [0.38; 0.78]	-0.039 [-0.07; -0.01]	$p = 2.00 \times 10^{-48}$; $p = 2.70 \times 10^{-34}$
Summer (Jun – Aug)	3.92 [3.70; 4.13]	-0.79 [-0.81; -0.75]	$p < 1 \times 10^{-308}$; $p < 1 \times 10^{-308}$
Fall (Sep – Nov)	3.51 [3.30; 3.69]	-0.73 [-0.75; -0.71]	$p < 1 \times 10^{-308}$; $p < 1 \times 10^{-308}$

Supplement E Longitudinal analysis

We conducted a longitudinal analysis among individuals who had at least one vitamin D measurement both before and during the pandemic. From the full dataset of repeated measurements, we identified a subset of $N = 61{,}393$ individuals with such longitudinal data. Specifically, we fitted a linear mixed-effects model to assess changes in vitamin D levels before and during the pandemic. The model included fixed effects for period, age group, gender, and season, and random intercepts and slopes for period at the individual level to account for repeated measurements. The results showed a significant increase in vitamin D levels during the pandemic compared to the pre-pandemic period ($\beta = 3.23$, SE $= 0.04$, $t = 74.94$, $p < 0.001$).

These findings contrast with our cross-sectional analyses, which consistently showed a decline in vitamin D levels during the pandemic. The results may reflect differences in population composition: the subset of individuals with repeated measurements may represent individuals with greater health awareness, better follow-up care, or targeted supplementation, leading to improved vitamin D status over time. In contrast, population-level trends captured in cross-sectional analyses are more likely to reflect broader behavioral or systemic shifts during the pandemic. We include this longitudinal analysis to complement our primary findings and provide additional nuance regarding within-individual changes over time.

[59] Austin, P. C. Balance diagnostics for comparing the distribution of baseline covariates between treatment groups in propensity-score matched samples. Statistics in Medicine 28, 3083–3107 (2009).

[60] R Core Team. R: A Language and Environment for Statistical Computing. R Foundation for Statistical Computing, Vienna, Austria (2023).

[61] Eurostat — GISCO, European Commission. Degree of urbanisation (2025). URL https://ec.europa.eu/eurostat/web/gisco/geodata/population-distribution/degree-urbanisation. Last accessed: 2025-07-12.
