# Supplementary material for: Monitoring changes in vitamin D levels during the COVID-19 pandemic with routinely-collected laboratory data
Source: Nat Commun. 2025 Oct 2;16:8772. doi: 10.1038/s41467-025-64192-6 (PMC12491433; doi:10.1038/s41467-025-64192-6)
Supplement: Supplementary file 2 — Reporting Summary [file 41467_2025_64192_MOESM2_ESM.pdf]

nature portfolio

Corresponding author(s): Eva Grill

Last updated by author(s): Aug 29, 2025

Reporting Summary

Nature Portfolio wishes to improve the reproducibility of the work that we publish. This form provides structure for consistency and transparency in reporting. For further information on Nature Portfolio policies, see our Editorial Policies and the Editorial Policy Checklist.

Statistics

For all statistical analyses, confirm that the following items are present in the figure legend, table legend, main text, or Methods section.

n/a	Confirmed	
☐	☒	The exact sample size (*n*) for each experimental group/condition, given as a discrete number and unit of measurement
☐	☒	A statement on whether measurements were taken from distinct samples or whether the same sample was measured repeatedly
☐	☒	The statistical test(s) used AND whether they are one- or two-sided<br>*Only common tests should be described solely by name; describe more complex techniques in the Methods section.*
☐	☒	A description of all covariates tested
☐	☒	A description of any assumptions or corrections, such as tests of normality and adjustment for multiple comparisons
☐	☒	A full description of the statistical parameters including central tendency (e.g. means) or other basic estimates (e.g. regression coefficient) AND variation (e.g. standard deviation) or associated estimates of uncertainty (e.g. confidence intervals)
☐	☒	For null hypothesis testing, the test statistic (e.g. *F*, *t*, *r*) with confidence intervals, effect sizes, degrees of freedom and *P* value noted<br>*Give P values as exact values whenever suitable.*
☒	☐	For Bayesian analysis, information on the choice of priors and Markov chain Monte Carlo settings
☒	☐	For hierarchical and complex designs, identification of the appropriate level for tests and full reporting of outcomes
☐	☒	Estimates of effect sizes (e.g. Cohen's *d*, Pearson's *r*), indicating how they were calculated

Our web collection on statistics for biologists contains articles on many of the points above.

Software and code

Policy information about availability of computer code

Data collection	no software was used
Data analysis	R (version 4.4.1), the R code used for the main analysis is retrievable at the public GitHub repository https://github.522 com/leaskapetze/vitamind_covid (DOI: 10.5281/zenodo.16980785)

For manuscripts utilizing custom algorithms or software that are central to the research but not yet described in published literature, software must be made available to editors and reviewers. We strongly encourage code deposition in a community repository (e.g. GitHub). See the Nature Portfolio guidelines for submitting code & software for further information.

Data

Policy information about availability of data

All manuscripts must include a data availability statement. This statement should provide the following information, where applicable:

- Accession codes, unique identifiers, or web links for publicly available datasets
- A description of any restrictions on data availability
- For clinical datasets or third party data, please ensure that the statement adheres to our policy

TThe data used in this study were licensed from Health Data Technologies GmbH ("Honic") and are subject to contractual restrictions. The de-identified patient-level data cannot be shared due to patient privacy consideration and licensing agreements. In accordance with the license agreements, access may be granted for the purpose of reviewing the study within a secure analytics environment, interested researchers may request access by contacting Honic (https://honic.eu/en/contact-

0). Requests will typically be reviewed within approximately 4-8 weeks. In accordance with data-sharing policies, only aggregated data can be made publicly available. For each figure, the corresponding aggregated data are provided in the source data file, with patient-level data reported exclusively as summary statistics.

Research involving human participants, their data, or biological material

Policy information about studies with human participants or human data. See also policy information about sex, gender (identity/presentation), and sexual orientation and race, ethnicity and racism.

Reporting on sex and gender	Information on gender was considered in study design. It was based on self-reporting (female: 186,711, male: 105,476). Gender-based analyses were reported. Participants who had self-identified as non-binary had to be excluded due to low sample size.
Reporting on race, ethnicity, or other socially relevant groupings	Data on race, ethnicity, or other socially relevant groupings was not available
Population characteristics	We analyzed routinely-collected laboratory data (N = 292,187 patients, 63.9% female,) from a large laboratory chain in Bavaria, Germany. Participants were adults 18 years and older at the time of vitamin D measurement. Due to reasons of anonymization and data protection, information on age was only available in broad age brackets. There is no information on diagnosis or genotype or therapy.
Recruitment	Data originated from a large laboratory chain within Bavaria/Germany comprising over 10 laboratories serving a wide range of medical practicioners. Patients are from both inpatient and outpatient settings, all types of health insurance, and a wide range of medical specializations. Data is representative for the German federal state of Bavaria. Selection bias is unlikely. Due to the automated electronic transfer of data, information bias because of transfer issues is unlikely. The laboratory chain has unified analysis protocols, so information bias due to methodological inconsistencies of sample processing and analysis is highly unlikely. As with all blood samples collected in routine care, the possibility of bias originating from sampling errors of the respective practicioners cannot be excluded.
Ethics oversight	The data request underwent a comprehensive assessment by an external compliance board, including patient and scientific representatives, to ensure the project met the ethical and legal standards required for using Honic data. This study received an Institutional Review Board exemption from the Ethics Committee of the Medical Faculty of LMU Munich (4-0900-KB).

Note that full information on the approval of the study protocol must also be provided in the manuscript.

Field-specific reporting

Please select the one below that is the best fit for your research. If you are not sure, read the appropriate sections before making your selection.

☒ Life sciences ☐ Behavioural & social sciences ☐ Ecological, evolutionary & environmental sciences

For a reference copy of the document with all sections, see nature.com/documents/nr-reporting-summary-flat.pdf

Life sciences study design

All studies must disclose on these points even when the disclosure is negative.

Sample size	Sample size was determined by using all available measurements within the given time frame (March 2018 to February 2022)
Data exclusions	Of 373,565 participants with at least one measurement, we excluded 81,378 participants due to one or several of the following pre-established exclusion criteria: (1) Participants were excluded if they did not have least one vitamin D measurement within the specified time frame (March 2018 to February 2022). (2) We excluded children and adolescents under 18 of age at the time of the vitamin D measurement as this was a precondition set by the compliance board. (3) We excluded participants, if they were not residing in Bavaria (defined by postal codes beginning with 60 or ranging from 80 to 97) to ensure a regionally homogeneous sample. (4) We excluded participants who had self-identified as non-binary because the group was too small for meaningful analysis.
Replication	Analysis code was re-checked, and results were replicated independently by a second researcher
Randomization	We compared measurements from two different time frames, pre-pandemic and pandemic. Participants were stratified by having their vitamin D status measured either in the pre-pandemic period (March 2018 to February 2020) or in the pandemic period (March 2020 to February 2022). To control for confounders, propensity score matching and causal random forest analyses were carried out.
Blinding	Investigators were not blinded for allocation during analysis, as patients were not actively allocated to groups.

Reporting for specific materials, systems and methods

We require information from authors about some types of materials, experimental systems and methods used in many studies. Here, indicate whether each material, system or method listed is relevant to your study. If you are not sure if a list item applies to your research, read the appropriate section before selecting a response.

Materials & experimental systems

n/a	Involved in the study
☒	☐ Antibodies
☒	☐ Eukaryotic cell lines
☒	☐ Palaeontology and archaeology
☒	☐ Animals and other organisms
☒	☐ Clinical data
☒	☐ Dual use research of concern
☒	☐ Plants

Methods

n/a	Involved in the study
☒	☐ ChIP-seq
☒	☐ Flow cytometry
☒	☐ MRI-based neuroimaging

Plants

Seed stocks	NA
Novel plant genotypes	NA
Authentication	NA
